# Efficacy and safety of pamidronate in Modic type 1 changes: study protocol for a prospective randomized controlled clinical trial

**DOI:** 10.1186/1745-6215-15-117

**Published:** 2014-04-09

**Authors:** Stella Cecchetti, Bruno Pereira, Antoine Roche, Christophe Deschaumes, Dihya Abdi, Emmanuel Coudeyre, Jean-Jacques Dubost, Sylvain Mathieu, Sandrine Malochet-Guinamand, Anne Tournadre, Marion Couderc, Marielle Vayssade, Coline Daron, Martin Soubrier

**Affiliations:** 1 Rheumatology Department, Clermont-Ferrand University Hospital, G Montpied Hospital, 58 Montalembert Street, F-63003 Clermont-Ferrand, France; 2 Faculty of Medicine, University of Clermont 1, 28 Henri Dunant Square, F-63001 Clermont-Ferrand, France; 3 Biostatistics Unit (Clinical Research and Innovation Department), Clermont-Ferrand University Hospital, 58 Montalembert Street, G Montpied Hospital, F-63003 Clermont-Ferrand, France; 4 Radiology Department A, Clermont-Ferrand University Hospital, G Montpied Hospital, 58 Montalembert Street, F-63003 Clermont-Ferrand, France; 5 Oral Surgery Department, Clermont-Ferrand University Hospital, 28 Henri Dunant Square, G Montpied Hospital, INSERM, U1107, F-63000 Clermont-Ferrand, France; 6 Physical Medicine and Rehabilitation Department, Clermont-Ferrand University Hospital, 58 Montalembert Street, G Montpied Hospital, F-63003 Clermont-Ferrand, France

**Keywords:** Modic type 1 changes, Chronic pain, Pamidronate, Randomized Controlled Trial

## Abstract

**Background:**

Erosive degenerative disc disease, also known as Modic type 1 changes, is usually characterized by low back pain with an inflammatory pain pattern, as seen in spondyloarthropathies. Intravenous pamidronate has proven to be effective in patients with ankylosing spondylitis who are refractory to nonsteroidal antiinflammatory drugs, and in painful bone diseases in general, such as Paget’s disease, fibrous dysplasia or vertebral fractures. We therefore hypothesize that pamidronate would be effective in treating low back pain associated with Modic type 1 changes.

**Methods/Design:**

This study, called PEPTIDE (short for the French title “Etude Prospective sur l’Efficacité et la tolérance du PamidronaTe dans les dIscopathies Degeneratives Erosives”), will be a double-blind, randomized, placebo-controlled, parallel group, phase two clinical trial. A total of 48 patients will be recruited. These patients will be randomly assigned to one of the two groups, with 24 patients in each group: one group will be given pamidronate and the other a placebo. Pamidronate will be administered at a dose of 90 mg per day for two days consecutively, and every patient, irrespective of treatment group, will be given paracetamol to maintain blinding by preventing drug-induced fever. The primary outcome measure is a between-group difference of 30 points on a 100 mm Visual Analogue Scale (VAS) at three months. Secondary outcome measures are improvement in functional status and the drug’s safety. Primary and secondary outcome measures will be assessed at each visit (inclusion, at six weeks, three months, and six months). If the primary goal is not attained, the patient will be offered a rigid or semi-rigid back brace, irrespective of the treatment group.

**Discussion:**

To date, only local treatments, for example intradiscal corticosteroid therapy, lumbar arthrodesis or back braces have been studied in randomized, controlled trials, with controversial results. This trial is currently ongoing and, if conclusive, should provide physicians with an acceptable alternative to those treatments. The results should be publicly available in spring 2015.

**Trial registration:**

ClinicalTrials.gov number, NCT01799616.

## Background

In 1988, Modic *et al.* suggested a classification scheme for endplate changes associated with erosive degenerative disk disease (EDDD) at the lumbar spine. Type 1 is characterized on a magnetic resonance imaging (MRI) scan by low signal on T1 and increased signal on T2 images, indicating bony edema [1]. EDDD is mostly a French denomination for a type of non-infectious erosive disease of the spine, among others, such as crystal deposition disease, erosive discopathy in patients under chronic hemodialyses and rheumatic diseases with inflammatory discopathy. EDDD is the degenerative, or osteoarthritic, subcategory of noninfectious erosive disk diseases. Erosions are not an essential feature of Modic type 1 changes, but the two have often been associated, since studies have described a constant presence of Modic type 1 changes in EDDD [2]. Moreover, usually superficial erosions have been described as one of the elementary bone lesions in degenerative disk disease associated with Modic type 1 changes [3]. Since ‘Modic type 1 changes’ is supposedly a radiological definition and does not encompass the clinical aspect, it has been accepted for a long time, in France at least, that we speak of EDDD, recently changed to ‘active discopathy’.

The correlation between Modic type 1 changes and back pain is still a matter of debate. There is however, to this date, far more evidence in favor of a real relationship between the two than otherwise. A 2008 systematic review [4] thus found ‘a positive association between vertebral endplate signal changes (VESC, or Modic changes), and low back pain (LBP) in the majority of studies reporting on this subject’, with Odds Ratios in the studies that reported a statistically significant positive association ranged between 2.0 and 19.9. Moreover, ‘a positive association between VESC and LBP has not only been found in the majority of studies of patients with LBP from different countries, but also in the general and working populations. In other words, there is a considerable consistency in this association’ [4].

Another 2008 review [5] confirmed the possibility of asymptomatic individuals with Modic changes but emphasized on its infrequency [6-8], and concluded that type 1 changes are ‘likely to be inflammatory in origin and seem to be strongly associated with active low back symptoms and segmental instability, thus reflecting a state of active degeneration and biomechanical instability of the lumbar spine’. For an MRI classification, Modic changes are therefore usually considered a validated clinical finding; Toyone *et al.* observed that 73% of patients with type 1 changes had low back pain, making asymptomatic subjects much less common than those with type 2 changes (11%) [6]. Modic type 1 changes have also been found to be specifically associated with the presence of low back pain [9], supporting the hypothesis that this early stage of the Modic disease is active [10]. Moreover, patients with Modic type 1 changes usually experience acute flares of previous common chronic lower back pain, usually with an inflammatory pattern [11]. Therefore, the clinical presentation of EDDDs mimics that of spondyloarthropathies (SpA), with consequent first-line treatment based on the use of nonsteroidal antiinflammatory drugs (NSAIDs) [12]. However, these treatments can be inadequate or even totally ineffective (at least 25% of patients are said to be refractory to NSAIDs), and sometimes poorly tolerated (gastroduodenal ulcers, arterial hypertension, and so forth) [13]. Corticosteroid therapy, which is not indicated for common low back pain, has been suggested for EDDDs by Maigne and Ballard, but over 50% of patients in the only open-label study experienced therapeutic failure [14].

Local treatments have been suggested, ranging from intradiscal cortisone injections to arthrodesis [15-20]. The results are, however, controversial [21,22]. These invasive procedures expose the patient to a risk of infection for intradiscal injections and to operating complications for arthrodesis [19].

Pamidronate, intravenous bisphosphonate with action on bone turnover, has shown some efficacy in open-label studies on spondyloarthropathies that are resistant to NSAIDs, whether on ankylosing spondylitis or SAPHO (Synovitis, Acne, Pustulosis, Hyperostosis, Osteitis) syndrome [22-31]. Although this efficacy in spondyloarthropathies remains a topic of discussion, it has nevertheless been clearly demonstrated for various types of bone pain (Paget’s disease [32], fibrous dysplasia of bone [33] and vertebral compression [34,35]). Therefore, we conducted an open-label study on the efficacy of pamidronate in ten patients with EDDD in 2006, with conclusive results [36]. At one year, four patients deemed their overall improvement to be excellent (85 to 100%), four to be good (70 to 85%) and two to be poor (< 55%). The inflammatory pain pattern disappeared as early as the first month in four patients and after one year in nine out of ten patients. Mean scores on the Visual Analogue Scale (VAS) showed gradual improvement, which was significant at six months (35.1 ± 30.2 mm; *p* = 0.05) and one year (32.1 ± 24 mm; *p* = 0.01). The improvement was quickly significant on the Quebec scale (a 20-item self-administered instrument designed to assess the level of functional disability in individuals with back pain), at one month (26 ± 27.7; *p* = 0.02) and at one year (15.8 ± 18; p = 0.001). The mean Schober score, measuring lumbar flexion mobility, was unchanged.

The objective of this clinical trial is to demonstrate the efficacy of pamidronate in degenerative disc diseases by conducting a prospective, randomized, placebo-controlled study. Patients with inflammatory low back pain and a Modic type 1 change on their MRI, whose inclusion and exclusion criteria are detailed in Table 1, will be randomly assigned to one of two groups: one group will be given pamidronate and the other placebo. Pamidronate will be administered at a dose of 90 mg per day for two days consecutively. The primary outcome measure is a between-group difference of 30 points on a 100 mm VAS at three months. Secondary outcome measures are improvement in functional status and the drug’s safety. In case of failure at three months, it will be recommended that the patient, irrespective of the treatment initially received, wear a rigid or semi-rigid dorsolumbar brace, whose efficacy will be evaluated using the same criteria at six months to ensure the patient is at least partially relieved at the end of the study, provided that they had not used a brace before the start of the study.

**Table 1:** Eligibility criteria

**Inclusion criteria**	**Exclusion criteria**
- Aged between 18 and 60 years	- Static disorders of the spine
- Low back pain with an inflammatory pattern (at least one of three characteristics: waking at night due to pain, morning stiffness for longer than 60 minutes, maximal pain on morning)	- Contraindication to pamidronate (hypocalcaemia, severe kidney failure or allergy)
	- Underage patients, patients subject to legal protections
	- Previous treatment with bisphosphonates
	- Pregnancy
- Daily pain for at least three months	
- VAS for pain > 40/100 in the last 48 hours	- Local or general infection
	- Previous disc surgery
- Lack of efficacy, intolerance, or contraindication to NSAIDs	
	- Systemic corticosteroid therapy in the last month
- Lack of efficacy of a rigid or semi-rigid back brace	- Epidural or facet joint corticosteroid injection in the last month
- Modic 1 disc disease (diagnosed on MRI and confirmed by a trained radiologist)	
	- History of septic spondylodiscitis
- Dental check-up within the last six months	- Ankylosing spondylitis
	- Low back pain associated with radiculalgia
- Signed informed consent form	- Active psychiatric disorder
	- Inability to read or understand French
	- Body temperature greater than 38°C (fever) or erythrocyte sedimentation rate greater than 20 mm/hour

MRI, magnetic resonance imaging; NSAIDs, non-steroidal anti-inflammatory drugs; VAS, Visual Analogic Scale.

## Methods and design

### Study design and location

PEPTIDE will be a double-blind, randomized, placebo-controlled on a 1:1 ratio, parallel group, phase two clinical trial. The endpoints will be measured at baseline, six weeks, three months and six months. In case of failure to alleviate pain with pamidronate at three months, a rigid back brace will be offered to all patients irrespective of the treatment group, and its efficacy will be assessed at six months. PEPTIDE will be conducted in the Rheumatology Department of Clermont-Ferrand University Hospital in France.

To maintain blinding, every patient regardless of the group they are in, will be given paracetamol to prevent pamidronate-induced fever insofar as is possible, and the product will only be labelled with the patient randomization number. Moreover, both the nurses and physicians from the rheumatology department responsible for administering the product and looking for side effects will be blinded to the participants’ status, and the assessments will be performed by physicians from the Physical Medicine and Rehabilitation Department who are unaware of said side effects.

### Participants

Participants are currently being recruited for this study from the Rheumatology and Physical Medicine and Rehabilitation Departments of Clermont-Ferrand University Hospital, and from the patients of local rheumatologists in private practice. The inclusion and exclusion criteria are described in Table 1.

### Approval

All patients will provide their informed consent and the study will be conducted according to the principles of the Declaration of Helsinki. This protocol has been approved by the Sud-Est VI Ethics Committee, which is affiliated with Clermont-Ferrand University Hospital, and by the French National Agency for Medicines and Health Products Safety (ANSM).

### Randomization

The randomization list will be drawn up by the methodologist in charge of the project before the start of the trial; this stratified randomization by sex and age (18 to 40 years, 40 to 50 years, and 50 to 60 years) will be carried out to balance the group sizes using a computer-generated allocation sequence with a block size of four; this will make it possible to monitor the eligibility of patients and transfer information on the randomization to the investigator, and possibly other correspondents.

### Description of the intervention

The study product is a 10 ml vial of disodium pamidronate 9 mg/ml, in a concentrated solution to be diluted in 500 ml of 0.9% sodium chloride solution, for slow intravenous infusion. It will be administered at the dose of 90 mg per day, for two days consecutively (for a total dose of 180 mg), as a slow intravenous infusion as in our pilot study [36]. We chose this administration pattern following Sayag-Boukris *et al.*[27] and Solau-Gervais *et al.*’s [31] method of treatment in SAPHO syndrome (60 mg each day, for three days consecutively), but divided the total dose of 180 mg between two days. We estimated that this would reduce the length of stay, and knew from our pilot study that 90 mg a day is usually well tolerated.

In the placebo group, the product used is a 500 ml bag of 0.9% sodium chloride solution.

In case of therapeutic failure at three months, the brace offered to patients will be a rigid or semi-rigid, lumbar or dorsolumbar brace custom made by the Physical Medicine and Rehabilitation Department.

### Outcome measures

The primary outcome measure will be the difference between D0 and D90 [16,20,22] in spinal pain assessed by VAS [36] between the treatment group and the placebo group (with a standard deviation of pain variation assessed by VAS set at 30 [19,36].

The secondary outcome measures will be the differences at three months between the treatment group and the placebo group in: the French adaptation of the Roland-Morris Low Back Pain Questionnaire (EIFEL), Dallas and Fear Avoidance Beliefs Questionnaire (FABQ) scores, the McMaster Toronto Arthritis Patient Preference Disability Questionnaire (MacTar) and the Minimum Clinically Important Improvement/Patient Acceptable Symptom State (MCII/PASS) questionnaire [37-40]; measurements of finger-to-floor distance and the Schober test; clinical signs of spinal inflammation, with assessment of the number of nighttime awakenings and the duration and severity of morning stiffness on a VAS; and safety, with the number and severity of side effects, physical or biological, such as hypocalcemia or renal toxicity by dosages of serum calcium and creatinine.

Outcomes will also be measured at six weeks as an interim evaluation, and at six months as follow up. Table 2 summarizes the data-collection schedule.

**Table 2:** Outcome measures at baseline, interim assessment, three months and follow-up

	**Baseline**	**Interim assessment -six weeks**	**Three months**	**Follow up -six months**
Informed consent	X			
Inclusion and exclusion criteria	X			
Demographic data	X			
Medical and surgical history	X			
Adverse effects		X	X	X
Current daily treatment	X	X	X	X
Dental status	X	X	X	X
Routine dental visit	X			
Physical examination	X	X	X	X
Blood test (CBC, hs-CRP, serum calcium, serum creatinine, ESR, vitamin D, PTH, CTX, osteocalcin)	X	X	X	X
Pain on 100 mm VAS	X	X	X	X
EIFEL score	X	X	X	X
FABQ score	X	X	X	X
Dallas score	X	X	X	X
MacTar score	X	X	X	X
MCII/PASS score		X	X	X
Finger-to-floor distance	X	X	X	X
Schober’s test	X	X	X	X
Number of nightly awakenings	X	X	X	X
Severity of MS (100 mm VAS)	X	X	X	X
Duration of MS (min)	X	X	X	X

CBC, complete blood count; CTX, C-terminal telopeptide; EIFEL: French adaptation of the Roland-Morris Low Back Pain Questionnaire; ESR: erythrocyte sedimentation rate; FABQ: Fear Avoidance Beliefs Questionnaire; hs-CRP: high-sensitivity C-reactive protein; MacTar: McMaster Toronto Arthritis Patient Preference Disability Questionnaire; MCII/PASS: Minimum Clinically Important Improvement/Patient Acceptable Symptom State; MS: morning stiffness; PTH: parathyroid hormone: VAS: visual analogue scale.

## Statistical considerations

### Sample size estimation

As the objective of this study is to show the efficacy of treatment with pamidronate on spinal pain attributed to Modic inflammatory disc disease, the justification of sample size estimation was based on the comparison of pain variation at three months, assessed using a VAS in the two randomization groups.

Therefore, considering type I and type II errors of 5% (two-sided) and 10%, respectively, 22 patients per group are needed to demonstrate a minimum difference of 30 points on the VAS scale of 0 to 100. As some subjects may be lost to follow up, 24 patients per group will be included.

### Statistical analysis

Intention-to-treat analysis will be conducted; all statistical tests will be considered for a type I error α of 5%.

Continuous variables will be presented as means and standard deviations, provided that their distribution is normal (Shapiro–Wilk test if needed [41]), or as medians, quartiles and extrema. Categorical parameters will be expressed as the number of subjects and associated percentages.

The patients will be described and compared between groups at inclusion using the following variables: fulfilment of eligibility criteria, epidemiological and clinical characteristics, and previous treatments.

As the primary outcome is variation in pain scored by the VAS, the randomization groups will be compared using a Student’s *t*-test or Mann–Whitney test if appropriate. Homoscedasticity will be tested using the Fisher–Snedecor test.

The between-group comparisons at 1.5, 3 and 6 months for the other outcome measures will be considered using standard tests, as has been described for the primary outcome measure of quantitative variables (notably EIFEL, Dallas, FABQ, finger-to-floor distance and Schober’s test), the Chi2 test or, where applicable, Fisher’s exact test for the qualitative variables. These between-group comparisons will be made systematically without adjustment, and by adjusting for factors whose distribution could be imbalanced between groups despite the randomization or due to clinical relevance (including analgesic use, treatments (and modifications) and other co-interventions during the study, such as physiotherapy). To measure the changes in the variables collected over the different visit time-points, a longitudinal data analysis will be conducted via ANOVA (ANalysis Of Variance) for repeated measures or the Friedman test (if ANOVA conditions are not fulfilled), followed by a post-hoc test and mixed models to take into account within- and between-patient variability. The intragroup comparisons, conducted secondarily, will be implemented via paired Student’s *t*-test or Wilcoxon test for the quantitative variables, and the Stuart–Maxwell test for the qualitative variables. Due to multiple comparisons envisaged at these three time-point evaluations, the inflation of type I error will be taken into account for each additional endpoint (Bonferroni adjustment should not be too conservative with only three time-points). The rate of subjects lost to follow up at three months is considered to be minor; the inclusion of 24 subjects is planned, while 22 patients should be necessary. In order to assess the problem caused by missing longitudinal data, estimation methods developed by Verbeke and Molenberghs [42] will be considered. For other missing data, if the frequency of missing data is >5%, an additional analysis will be performed using the multiple imputation method (Stata’s mi command software [43]).

## Discussion

The treatment options for Modic type 1 disc diseases remain quite limited; invasive local treatments, such as intradiscal injections of cortisone derivatives or spinal arthrodesis, have generated controversial results [21,22], and the brace remains a last-resort solution for patients who wish to maintain some mobility and often consider it cumbersome and unsightly.

There are, to date, no published controlled trials involving pamidronate in EDDD. The results from our pilot study (an open-label study with ten subjects) were encouraging [36], and therefore we hypothesized, in light of this pilot study and the widespread use of pamidronate in painful bone diseases, that pamidronate could be effective in relieving EDDD-induced pain. However, the efficacy of pamidronate in this condition clearly required further investigation in a prospective, blinded, randomized controlled trial. Subject recruitment began in January 2013 and will continue until the end of December 2014, with the results made available in March 2015.

Regarding safety concerns, vitamin D blood levels will be measured before the product is administered and patients will be given vitamin D supplementation accordingly prior to treatment [44]. Every patient will also be examined by the dentist affiliated with the study to look for, and if necessary treat, contraindications to intravenous bisphosphonates.

All studies include biases, and we have taken several steps to limit those that could arise in this study; as pamidronate can cause fever and flu-like syndromes, the patient will be included by one physician and evaluated by another. In addition, to minimize this effect, patients from both groups will receive 1 g of paracetamol prior to the infusion and 1 g of paracetamol every 8 hours for 48 hours. To guarantee the double-blind and prevent biases in monitoring and measurement, physicians from the Rheumatology Department will be responsible for administering the product and monitoring the side effects (such as hyperthermia in cases of patients receiving pamidronate despite the administration of paracetamol, which would result in unblinding for both the patient and physician), and physicians, physiotherapists and occupational therapists from the Physical Medicine and Rehabilitation Department will be responsible for assessments (D0, D45, D90 and D180). In addition, and again to guarantee the blinding for the patient, the products administered intravenously will be identified not by the product name but by the patient’s randomization number, and will be prepared by the hospital pharmacy so that the nurses performing the infusions will not know which product is infused to the patient. Treatment changes over the six months of follow up will be recorded and, to prevent a recall bias, each patient will be given a diary including a table to be completed following each change in his or her standard treatment. Finally, an experienced radiologist affiliated with the study and specializing in osteoarticular diseases is responsible for confirming each radiological diagnosis of Modic type 1 disc disease on an MRI scan. Concerning the sample size estimation, the assumption of our protocol is that the standard-deviation of the difference (test versus control) in change scores (initial versus final) is the same as the previous standard-deviation of the change score found for treated patients in the open pilot study [36]. Therefore, sample size estimation is based on previous works, which could be considered heterogeneous for the standard-deviation of the change score. In our pilot study [36], the standard-deviation of VAS was 19.1 at baseline, 22.7 at three months and 24.1 for change score. As standard-deviation equals 30, our estimation appears near to the expected results and overestimates the value obtained in the pilot study. Nevertheless, blinded studies have a smaller effect which may not be captured by the standard-deviation. Moreover, the statistical power was voluntarily more important than 80% (1-β fixed equals 90%). Concerning the statistical approach, the primary outcome analysis will be pain-VAS difference at three months using a Student’s *t*-test if normally distributed and a Mann–Whitney test if not. In the secondary outcome analysis, a multivariate situation should be proposed using a linear regression model to take adjustment factors into account. Adjustments may threaten the randomized nature of the study. To further ensure the randomization of the study, a random-effect model, considering block as a random effect could be proposed.

## Trial status

PEPTIDE is ongoing and currently recruiting.

## Abbreviations

ANOVA: ANalysis Of Variance; CBC: complete blood count; CTX: C-terminal telopeptide; EDDD: erosive degenerative disc disease; EIFEL: French adaptation of the Roland-Morris Low Back Pain Questionnaire; ESR: erythrocyte sedimentation rate; FABQ: Fear Avoidance Beliefs Questionnaire; hs-CRP: high-sensitivity C-reactive protein; LBP: low back pain; MacTar: McMaster Toronto Arthritis Patient Preference Disability Questionnaire; MCII/PASS: Minimum Clinically Important Improvement/Patient Acceptable Symptom State; MRI: magnetic resonance imaging; MS: morning stiffness; NSAIDs: nonsteroidal antiinflammatory drugs; PEPTIDE: short for the French title “Etude Prospective sur l’Efficacité et la tolérance du PamidronaTe dans les dIscopathies Degeneratives Erosives”; PTH: parathyroid hormone; SAPHO: Synovitis Acne Pustulosis Hyperostosis Osteitis; VAS: visual analogue scale; VESC: vertebral endplate signal changes.

## Competing interests

The authors declare that they have no conflicts of interest.

## Authors’ contributions

MS: conception and design, data collection, analysis and interpretation, manuscript writing, critical revision and final approval of the manuscript. SC: conception and design, data collection, analysis and interpretation, manuscript writing, critical revision and final approval of the manuscript. DA: design, data collection and analysis, critical revision and final approval of the manuscript. BP: conception, data analysis, manuscript writing, critical revision and final approval of the manuscript. CDa: data collection, critical revision and final approval of the manuscript. MV: data collection, critical revision and final approval of the manuscript. EC: design, data collection, critical revision and final approval of the manuscript. SM: conception and design, data collection, critical revision and final approval of the manuscript. SMG: data collection, critical revision and final approval of the manuscript. AT: data collection, critical revision and final approval of the manuscript. MC: data collection, critical revision and final approval of the manuscript. JJD: data collection, critical revision and final approval of the manuscript. AR: data analysis, critical revision and final approval of the manuscript. CDe: data collection, critical revision and final approval of the manuscript. All authors read and approved the final manuscript.
